# 

**Published:** 1900-03

**Authors:** 


					﻿This Journal and Atlanta Semi-weekly Journal one year for $1.00
cash in advance. No checks.
This Journal and Atlanta Semi-weekly Journal one year for $1.00
cash in advance. No checks.
This Journal and Atlanta Semi-weekly Journal one year for $1.00
cash in advance. No checks.
This Journal and Atlanta Semi-weekly Journal one year for $1.00
cash in advance. No checks.
This Journal and Atlanta Semi-weekly Journal one year for $1.00
cash in advance. No checks.
This Journal and Atlanta Semi-weekly Journal one year for $1 00
cash in advance. No checks.
This Journal and Atlanta Semi-weekly Journal one year for $1.00
cash in advance. No checks.
				

